# Supplementary material for: Newly incident cannabis use in the United States, 2002–2011: a regional and state level benchmark
Source: PeerJ. 2017 Jul 25;5:e3616. doi: 10.7717/peerj.3616 (PMC5530998; doi:10.7717/peerj.3616)
Supplement: Table S1 — Footnotes: (1) Our research team is responsible for the calculations and estimates for 12-to-24-year-olds as shown in this table. We produced these estimates using the R-DAS public use datasets, which made it possible to focus on the 12-to-24-year old population subgroup that previously has been documented as an exceptionally high risk adolescent-to-young-adult interval for starting to use cannabis, as compared with older adult subgroups. We utilized the 2002–2011 ten-year NSDUH set to produce the cannabis incidence estimates shown here. We constrained the precision of the estimates (i.e., the number of units to the right of the decimal point) based upon the fact that state-specific samples, in aggregate, tended to be in a range from the 100s to 1,000 s of survey participants. That is, it did not make sense to produce an estimate to the 10,000ths place when the number in the state-specific sample was well under 10,000 persons. (2) In order to produce the cannabis incidence estimates for the US population age 12 years and older, we turned to an online analysis ‘calculator’ created by the US government in 2016–17, from which it has been possible to derive cannabis incidence estimates for the 51 jurisdictions under study, for the community population age 12 years and older, along with corresponding 95% confidence intervals. The documentation on methods used to produce these incidence estimates suggests that a pooled analysis approach has been used, as opposed to the meta-analysis approach we chose, but the documentation is not fully transparent with respect to these methodological details. For this reason, we have not included these estimates in our primary report. Instead, we offer them to interested readers, and must leave the issues of interpretation up to those readers. In this instance, the online ‘calculator’ provides state-level estimates much more precise than the estimates we can support. Interested readers should consider whether the available information abo [file peerj-05-3616-s001.docx]

|  | | | | |  |
| --- | --- | --- | --- | --- | --- |
| State | 2002-2011  Estimated  Incidence  Age 12-24 | 95% CI | 2010-2011  Estimated  Incidence  Age 12 and older | 95% CI | |
| Alabama | 4 | 4, 5 | 1.39 | 1.19, 1.63 | |
| Alaska | 6 | 5, 7 | 2.88 | 2.46, 3.37 | |
| Arizona | 5 | 5, 7 | 1.97 | 1.69, 2.29 | |
| Arkansas | 5 | 4, 5 | 1.51 | 1.29, 1.76 | |
| California | 6 | 6, 6 | 2.25 | 2.06, 2.46 | |
| Colorado | 8 | 7, 9 | 2.75 | 2.36, 3.21 | |
| Connecticut | 7 | 6, 9 | 2.01 | 1.76, 2.37 | |
| District of Columbia | 7 | 6, 8 | 2.98 | 2.52, 3.51 | |
| Delaware | 7 | 6, 8 | 2.29 | 1.97, 2.65 | |
| Florida | 6 | 5, 6 | 1.50 | 1.37, 1.65 | |
| Georgia | 5 | 4, 6 | 1.76 | 1.52, 2.05 | |
| Hawaii | 6 | 5, 7 | 2.01 | 1.68, 2.39 | |
| Idaho | 5 | 4, 6 | 1.93 | 1.66, 2.24 | |
| Illinois | 5 | 5, 6 | 1.85 | 1.69, 2.03 | |
| Indiana | 6 | 5, 6 | 1.73 | 1.50, 1.99 | |
| Iowa | 5 | 4, 6 | 1.75 | 1.50, 2.05 | |
| Kansas | 5 | 4, 6 | 1.85 | 1.59, 2.15 | |
| Kentucky | 5 | 4, 6 | 1.52 | 1.31, 1.77 | |
| Louisiana | 5 | 4, 6 | 1.42 | 1.22, 1.65 | |
| Maine | 7 | 6, 8 | 2.02 | 1.74, 2.35 | |
| Maryland | 6 | 5, 7 | 1.71 | 1.47, 1.99 | |
| Massachusetts | 8 | 7, 9 | 2.50 | 2.16, 2.90 | |
| Michigan | 6 | 6, 7 | 2.14 | 1.96, 2.34 | |
| Minnesota | 5 | 5, 6 | 1.83 | 1.58, 2.12 | |
| Mississippi | 3 | 3, 4 | 1.46 | 1.25, 1.70 | |
| Missouri | 5 | 5, 6 | 1.58 | 1.36, 1.84 | |
| Montana | 8 | 7, 9 | 2.80 | 2.44, 3.21 | |
| Nebraska | 5 | 4, 6 | 1.59 | 1.36, 1.86 | |
| Nevada | 6 | 5, 7 | 2.12 | 1.81, 2.49 | |
| New Hampshire | 7 | 6, 8 | 2.61 | 2.24, 3.04 | |
| New Jersey | 6 | 5, 6 | 1.91 | 1.64, 2.22 | |
| New Mexico | 7 | 6, 8 | 2.40 | 2.08, 2.78 | |
| New York | 6 | 6, 7 | 1.78 | 1.63, 1.95 | |
| North Carolina | 5 | 5, 6 | 1.87 | 1.61, 2.16 | |
| North Dakota | 5 | 5, 6 | 1.72 | 1.47, 2.01 | |
| Ohio | 6 | 5, 6 | 1.85 | 1.69, 2.02 | |
| Oklahoma | 5 | 4, 6 | 1.63 | 1.40, 1.91 | |
| Oregon | 7 | 6, 9 | 2.20 | 1.90, 2.53 | |
| Pennsylvania | 6 | 6, 7 | 1.80 | 1.64, 1.98 | |
| Rhode Island | 8 | 6, 9 | 2.49 | 2.14, 2.90 | |
| South Carolina | 5 | 4, 5 | 1.63 | 1.41, 1.88 | |
| South Dakota | 6 | 5, 7 | 1.60 | 1.36, 1.88 | |
| Tennessee | 4 | 3, 4 | 1.50 | 1.28, 1.75 | |
| Texas | 5 | 4, 5 | 1.82 | 1.66, 2.00 | |
| Utah | 3 | 3, 4 | 1.37 | 1.14, 1.64 | |
| Vermont | 9 | 8, 10 | 2.72 | 2.34, 3.17 | |
| Virginia | 6 | 5, 7 | 1.91 | 1.64, 2.21 | |
| Washington | 6 | 5, 7 | 2.23 | 1.92, 2.59 | |
| West Virginia | 5 | 4, 6 | 1.64 | 1.42, 1.89 | |
| Wisconsin | 6 | 6, 7 | 1.53 | 1.29, 1.81 | |
| Wyoming | 6 | 5, 7 | 1.80 | 1.55, 2.09 | |
