# Supplementary material for: Newly incident cannabis use in the United States, 2002–2011: a regional and state level benchmark
Source: PeerJ. 2017 Jul 25;5:e3616. doi: 10.7717/peerj.3616 (PMC5530998; doi:10.7717/peerj.3616)
Supplement: Table S2 — Data from United States National Surveys on Drug Use and Health for the years 2002–2011. [file peerj-05-3616-s002.docx]

| **Year** | **Region** | | | | **Age 12-17** | **Age 18-25** | **Age 26+** | **Male** | **Female** |
| --- | --- | --- | --- | --- | --- | --- | --- | --- | --- |
|  | **Northeast** | **Midwest** | **South** | **West** |  |  |  |  |  |
| **2002** |  |  |  |  |  |  |  |  |  |
| Selected Persons | 16,490 | 22,588 | 24,530 | 16,973 | 26,230 | 27,216 | 27,135 | 39,453 | 41,128 |
| Completed Interviews | 13,706 | 19,180 | 20,900 | 14,340 | 23,659 | 23,271 | 21,196 | 32,766 | 35,360 |
| Weighted Response Rate | 75.57% | 80.01% | 79.99% | 77.33% | 89.99% | 85.16% | 75.81% | 77.06% | 79.99% |
| **2003** |  |  |  |  |  |  |  |  |  |
| Selected Persons | 16,736 | 22,665 | 24,725 | 17,505 | 25,387 | 27,259 | 28,985 | 40,008 | 41,623 |
| Completed Interviews | 13,655 | 18,993 | 20,612 | 14,524 | 22,696 | 22,941 | 22,147 | 32,627 | 35,157 |
| Weighted Response Rate | 75.20% | 78.56% | 78.38% | 76.51% | 89.57% | 83.47% | 74.63% | 75.72% | 78.96% |
| **2004** |  |  |  |  |  |  |  |  |  |
| Selected Persons | 16,674 | 22,920 | 24,820 | 17,559 | 25,141 | 27,408 | 29,424 | 40,194 | 41,779 |
| Completed Interviews | 13,523 | 18,889 | 20,807 | 14,541 | 22,309 | 23,075 | 22,376 | 32,697 | 35,063 |
| Weighted Response Rate | 75.14% | 77.63% | 78.65% | 75.38% | 88.56% | 83.87% | 74.22% | 75.44% | 78.46% |
| **2005** |  |  |  |  |  |  |  |  |  |
| Selected Persons | 16,994 | 23,542 | 25,411 | 17,858 | 25,840 | 27,337 | 30,628 | 41,054 | 42,751 |
| Completed Interviews | 13,711 | 19,154 | 20,818 | 14,625 | 22,565 | 22,764 | 22,979 | 32,787 | 35,521 |
| Weighted Response Rate | 73.66% | 76.42% | 77.16% | 76.42% | 87.10% | 83.06% | 73.50% | 74.45% | 77.80% |
| **2006** |  |  |  |  |  |  |  |  |  |
| Selected Persons | 17,201 | 23,766 | 25,848 | 18,219 | 26,702 | 27,303 | 31,029 | 41,833 | 43,201 |
| Completed Interviews | 13,499 | 18,988 | 20,841 | 14,474 | 22,912 | 22,152 | 22,738 | 32,696 | 35,106 |
| Weighted Response Rate | 71.96% | 75.39% | 75.13% | 73.60% | 85.46% | 80.96% | 71.54% | 72.44% | 75.92% |
| **2007** |  |  |  |  |  |  |  |  |  |
| Selected Persons | 17,486 | 24,150 | 25,737 | 18,401 | 26,191 | 28,085 | 31,498 | 42,280 | 43,494 |
| Completed Interviews | 13,642 | 19,110 | 20,683 | 14,435 | 22,475 | 22,409 | 22,986 | 32,802 | 35,068 |
| Weighted Response Rate | 71.65% | 74.34% | 75.75% | 72.52% | 85.35% | 79.76% | 71.42% | 72.06% | 75.69% |
| **2008** |  |  |  |  |  |  |  |  |  |
| Selected Persons | 17,336 | 24,383 | 25,641 | 19,075 | 26,501 | 29,091 | 30,843 | 42,460 | 43,975 |
| Completed Interviews | 13,594 | 19,314 | 20,877 | 14,951 | 22,559 | 23,468 | 22,709 | 33,120 | 35,616 |
| Weighted Response Rate | 72.48% | 74.93% | 76.59% | 72.24% | 84.73% | 80.67% | 72.00% | 72.39% | 76.37% |
| **2009** |  |  |  |  |  |  |  |  |  |
| Selected Persons | 17,503 | 23,827 | 25,560 | 18,539 | 26,377 | 28,444 | 30,608 | 42,008 | 43,421 |
| Completed Interviews | 13,772 | 19,133 | 20,976 | 14,819 | 22,644 | 23,248 | 22,808 | 33,282 | 35,418 |
| Weighted Response Rate | 73.44% | 75.97% | 77.39% | 74.50% | 85.73% | 81.70% | 73.34% | 74.21% | 77.07% |
| **2010** |  |  |  |  |  |  |  |  |  |
| Selected Persons | 16,782 | 24,139 | 25,597 | 18,479 | 25,908 | 28,164 | 30,925 | 41,782 | 43,215 |
| Completed Interviews | 13,017 | 19,301 | 20,769 | 14,717 | 21,992 | 23,026 | 22,786 | 32,826 | 34,978 |
| Weighted Response Rate | 72.81% | 74.81% | 76.24% | 73.17% | 84.65% | 81.20% | 72.14% | 73.11% | 75.94% |
| **2011** |  |  |  |  |  |  |  |  |  |
| Selected Persons | 17,251 | 24,570 | 28,122 | 18,593 | 27,911 | 28,589 | 32,036 | 43,436 | 45,100 |
| Completed Interviews | 13,090 | 19,258 | 22,980 | 14,781 | 23,549 | 23,083 | 23,477 | 33,779 | 36,330 |
| Weighted Response Rate | 69.86% | 73.92% | 76.88% | 74.41% | 84.95% | 80.48% | 71.96% | 72.49% | 76.14% |
