# Supplementary material for: Newly incident cannabis use in the United States, 2002–2011: a regional and state level benchmark
Source: PeerJ. 2017 Jul 25;5:e3616. doi: 10.7717/peerj.3616 (PMC5530998; doi:10.7717/peerj.3616)
Supplement: Table S3 — Footnote: The individual z-tests are based on a null hypothesis that the two sample proportions are equal with a significance level of 0.05. Each state SE was taken from the R-DAS output and the region SE was calculated as follows: SEregion = √((SEstate1)2 + (SE state2)2 + ⋯ + (SEstaten)2). The pooled SE was a similar combination of the Mexico border SE and each comparative region SE using SEpooled = √((SEmexico)2 + (SE region)2). In each calculation of combined SE, the covariance term is ignored based on the assumption that samples are from independent populations of separate states. We calculated the z-statistics such that: z-statistic $=(p{\widehat{}}_{\mathrm{mexico}}-p{\widehat{}}_{\mathrm{region}})/{\mathrm{SE}}_{\mathrm{pooled}}$=p ^mexico−p ^region∕SEpooled using the proportions ($p\widehat{}$p ^) from the raw data region estimates shown in Fig. 1. The z-statistics all led to a corresponding p-value and a result of keeping the null, because all p-values were greater than 0.05. [file peerj-05-3616-s003.docx]

| Region being compared to the Mexico Border: | SE_region_ | SE_pooled_ | p̂_region_ | z-statistic | p-value |
| --- | --- | --- | --- | --- | --- |
| Mexico Border | 0.007607 | - | 0.05486 | - | - |
| Pacific Border | 0.006269 | 0.009857 | 0.06513 | -1.04 | 0.1492 |
| Gulf of Mexico Border | 0.006635 | 0.010094 | 0.04605 | 0.87 | 0.1922 |
| Non-Border | 0.016702 | 0.018352 | 0.05256 | 0.13 | 0.4483 |
| North Atlantic Border | 0.012456 | 0.014595 | 0.06896 | -0.97 | 0.1660 |
| South Atlantic Border | 0.008674 | 0.011537 | 0.05229 | 0.22 | 0.4129 |
| Canada Border | 0.015554 | 0.017315 | 0.06245 | -0.44 | 0.3300 |
| Washington | 0.004539 | 0.008858 | 0.05943 | -0.52 | 0.3015 |
| Alaska | 0.005161 | 0.009193 | 0.05858 | -0.40 | 0.3446 |
| Hawaii | 0.005764 | 0.009544 | 0.06028 | -0.57 | 0.2843 |
