# Supplementary material for: Newly incident cannabis use in the United States, 2002–2011: a regional and state level benchmark
Source: PeerJ. 2017 Jul 25;5:e3616. doi: 10.7717/peerj.3616 (PMC5530998; doi:10.7717/peerj.3616)
Supplement: Figure S1 — Data from United States National Surveys on Drug Use and Health 10-Year Restricted Data Analysis System, 2002–2011 (Unweighted n ∼ 420, 000 12–24 year olds). [file peerj-05-3616-s004.docx]

|  | |
| --- | --- |
| Canada Border States | Non-Border States |
| 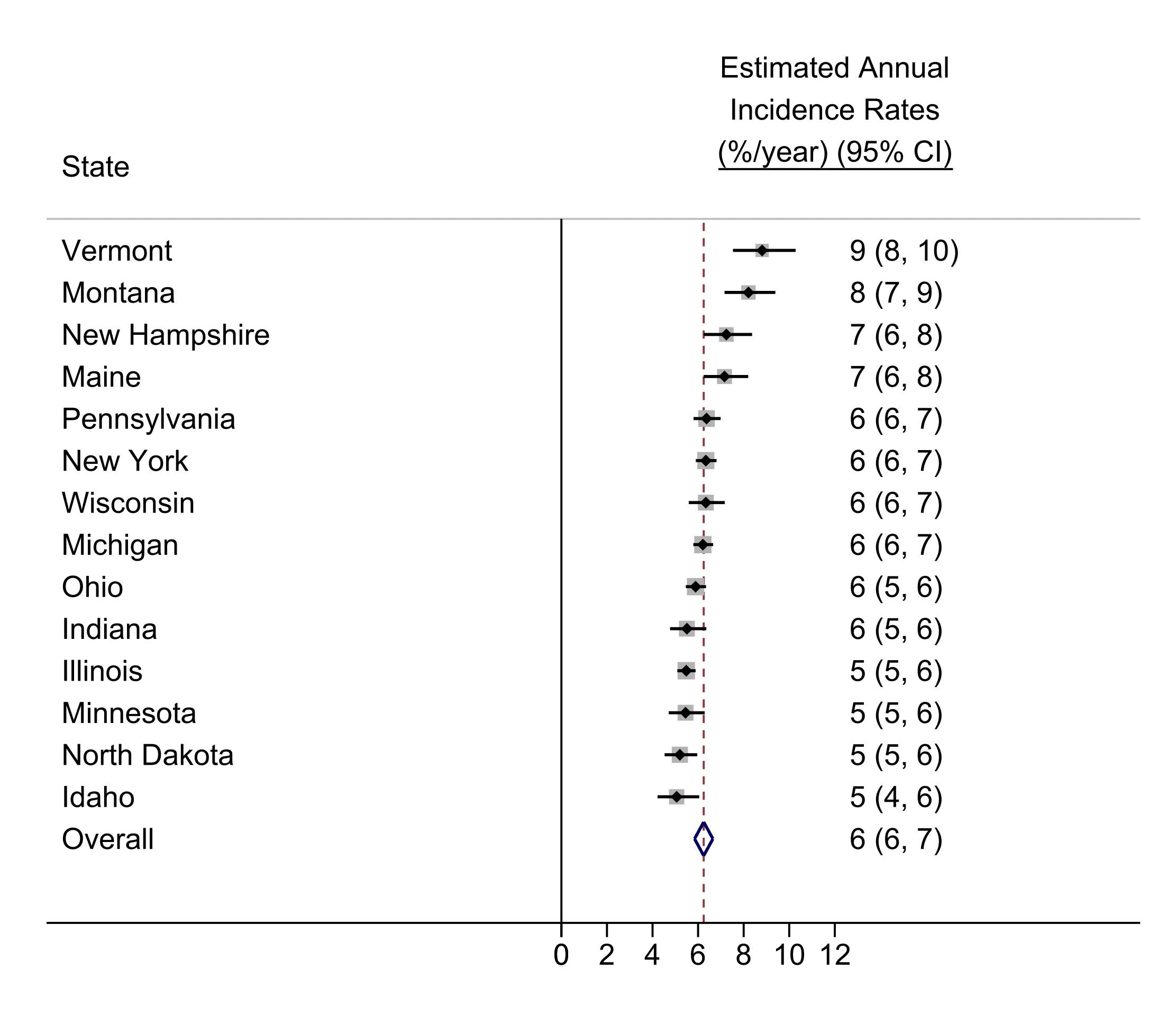  | 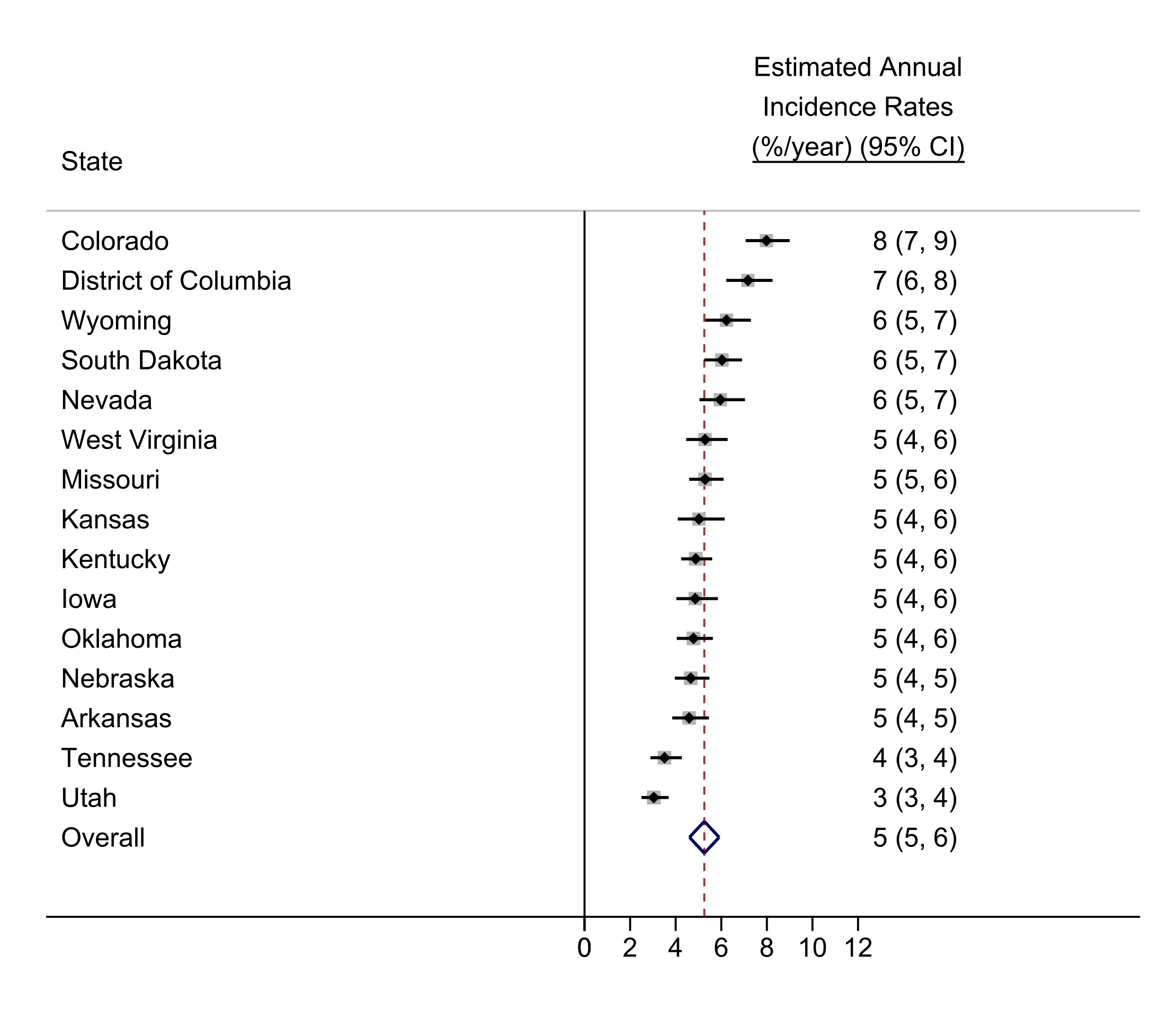 |

| Gulf of Mexico Border | South Atlantic Border |
| --- | --- |
| 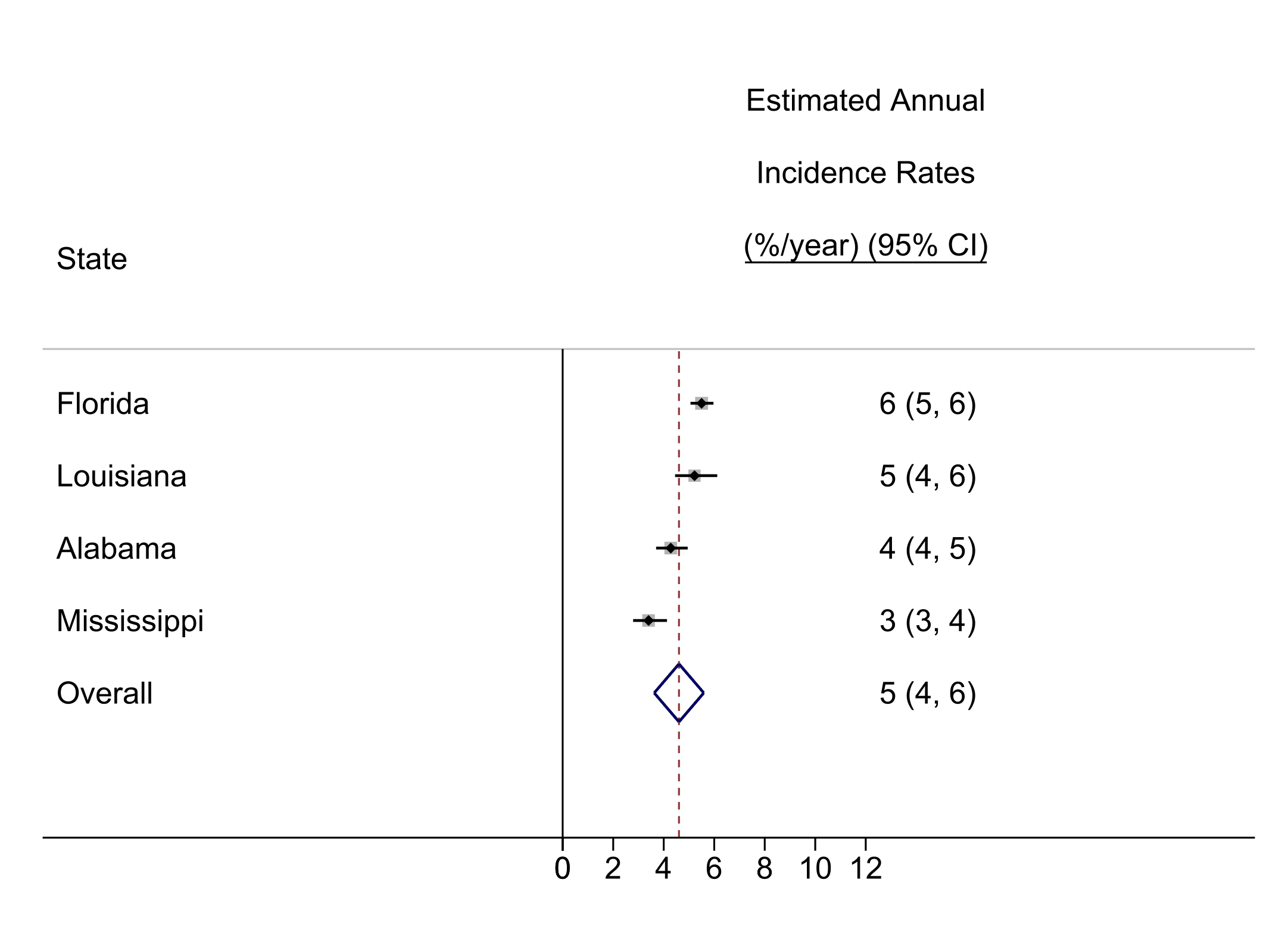 | 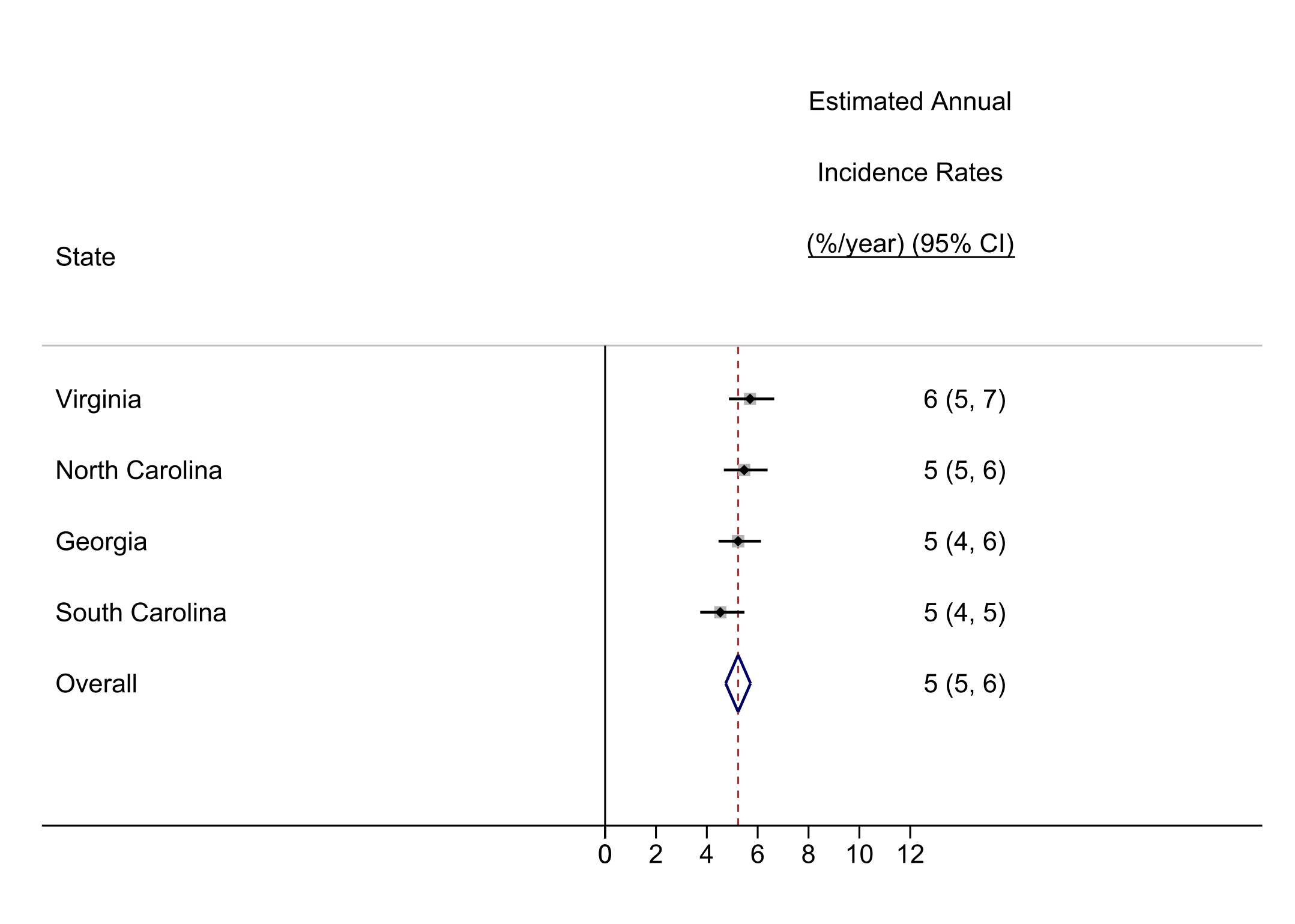 |

| Mexico Border | Pacific Border |
| --- | --- |
| **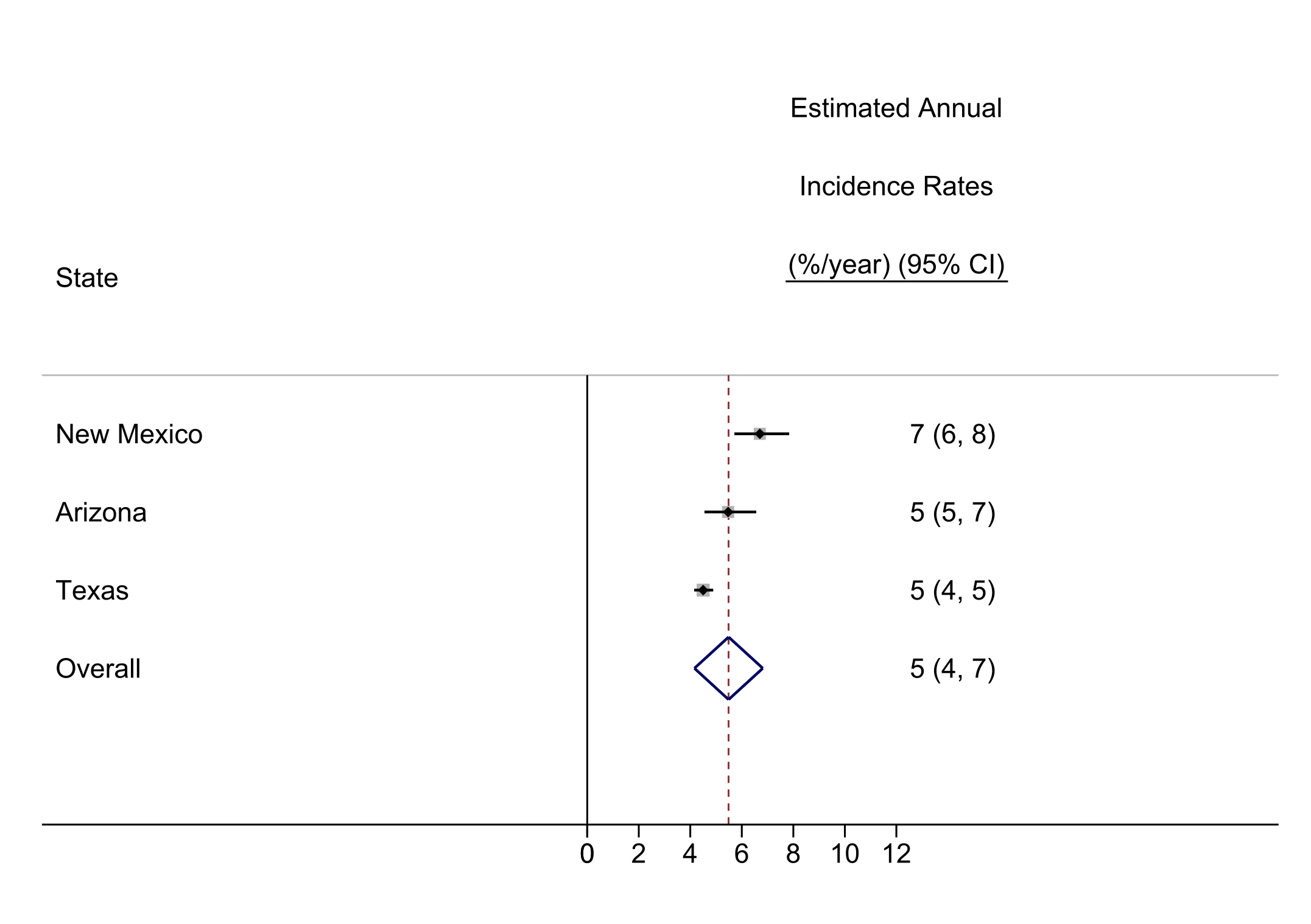** | 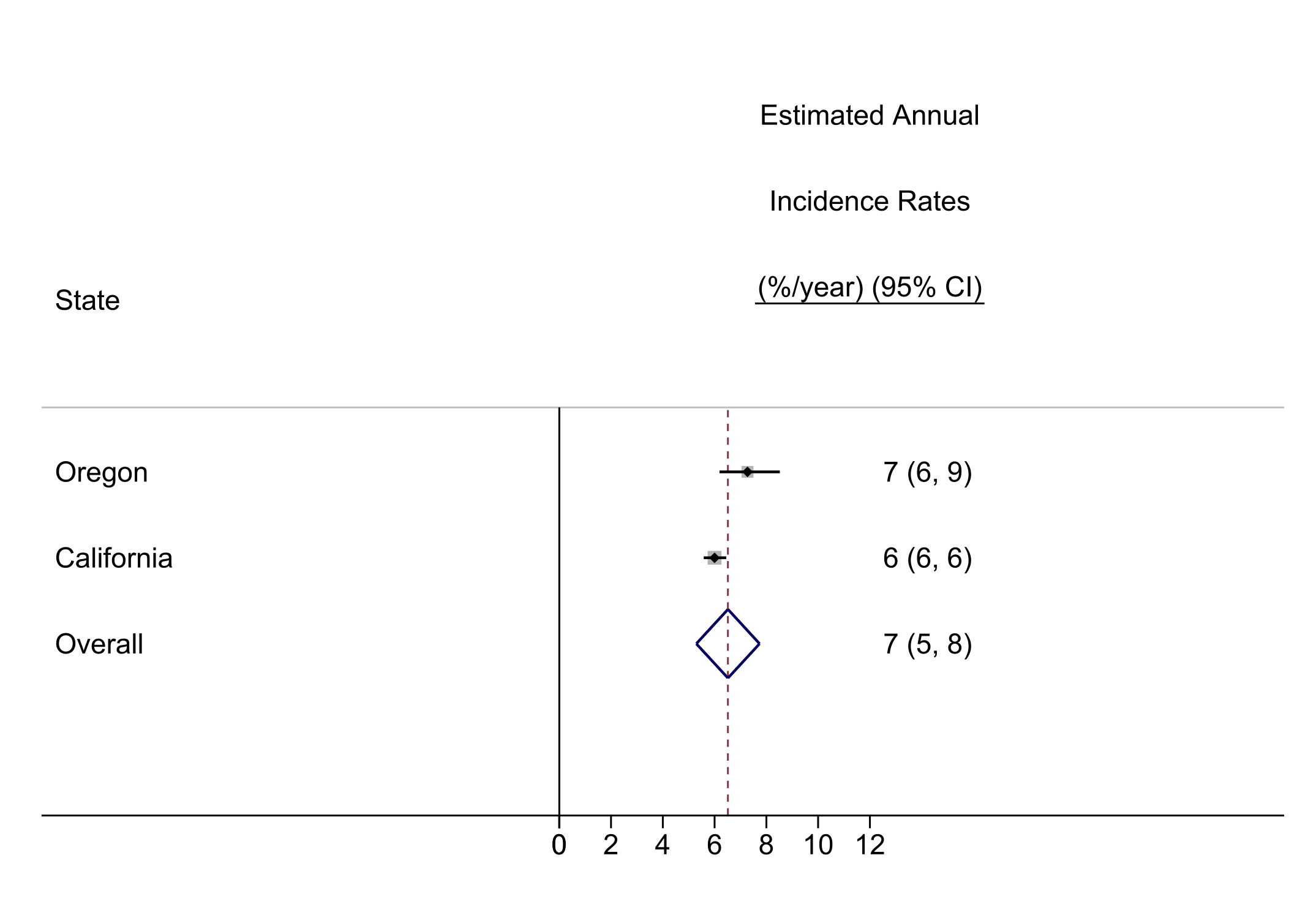 |

| North Atlantic Border |
| --- |
| 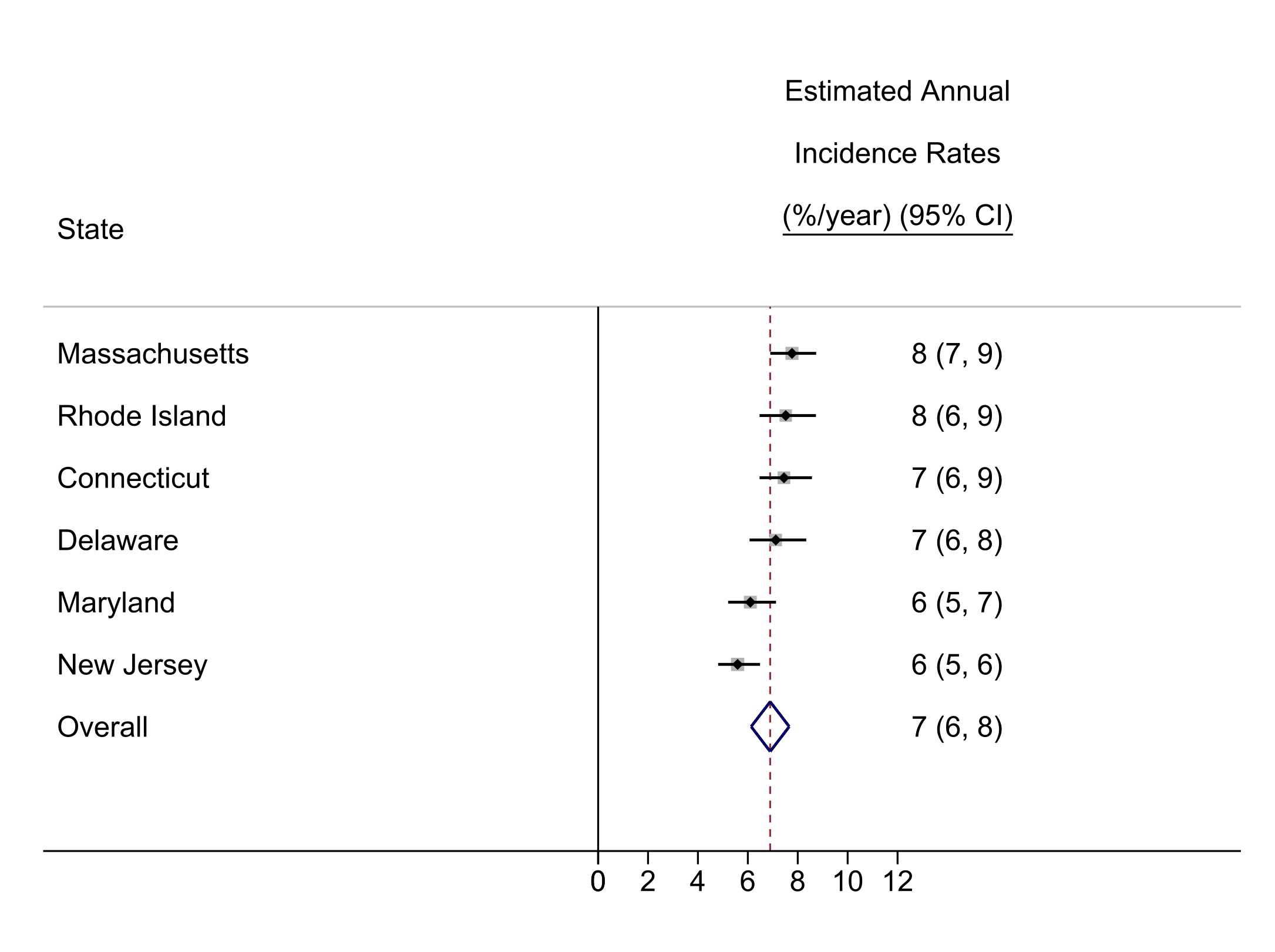 |
